# The genome sequence of the grey top shell, *Steromphala cineraria *(Linnaeus, 1758)

**DOI:** 10.12688/wellcomeopenres.17677.2

**Published:** 2022-03-24

**Authors:** Patrick Adkins, Robert Mrowicki, Joanna Harley, Nova Mieszkowska, João G. R. N. Ferreira, Marine Biological Association Genome Acquisition Lab, Darwin Tree of Life Barcoding collective, Wellcome Sanger Institute Tree of Life programme, Wellcome Sanger Institute Scientific Operations: DNA Pipelines collective, Tree of Life Core Informatics collective, Darwin Tree of Life Consortium

**Affiliations:** 1 Marine Biological Association, Plymouth, Devon, UK; 2 Natural History Museum, London, UK; 3 Bio Bureau Biotechnology, Rio de Janeiro, Brazil; 4 Tree of Life, Wellcome Sanger Institute, Cambridge, UK

**Keywords:** Steromphala cineraria, grey topshell, genome sequence, chromosomal, Mollusca

## Abstract

We present a genome assembly from an individual *Steromphala cineraria* (the grey topshell; Mollusca; Gastropoda; Trochida; Trochidae). The genome sequence is 1,270 megabases in span. The majority of the assembly (99.23%) is scaffolded into 18 chromosomal pseudomolecules.

## Species taxonomy

Eukaryota; Metazoa; Spiralia; Lophotrochozoa; Mollusca; Gastropoda; Vetigastropoda; Trochida; Trochoidea; Trochidae; Cantharidinae; Steromphala; *Steromphala cineraria* (Linnaeus, 1758) (NCBI:txid216125).

## Background

*Steromphala cineraria* (Linnaeus, 1758), commonly called the grey topshell, is a gastropod common to rocky shores in the UK. It typically occurs among boulders and cobbles on the lowshore and sub-tidally, where it grazes among *Fucus* and *Laminaria* species. Intertidally, it is most common on the lower shore, but can also be found in pools higher on the shore. Sub-tidally it extends to depths of 130 m, although it is most common in the kelp forests between 30 m and low water spring tide ( Fretter & Graham, 1976). Its geographical distribution ranges from southern Portugal and north to the White Sea in northern Russia, becoming rarer at its range edges as thermal limits are approached ( Nekhaev, 2013).

An important grazing species, *S. cineraria* is distinguished from other species of trochids by its bluntly conical shell and grey/yellowish finely striped patterning on the shell. In smaller shells, the umbilicus is large, becoming smaller and elliptical with age and in large shells sometimes becoming overgrown by the columellar lip ( Fretter & Graham, 1976).

As *S. cineraria* is found across a large range of latitudes, it is exposed to a wide range of thermal environments in temperature, both due to time of year and geographical distribution. It is important to understand how populations may change in response to climate change, especially in its southern and northern range limits, and the knock-on effects this may have on macroalgae due to changes in grazing populations ( Mieszkowska *et al.*, 2007). A high quality genome sequence for this species will allow future studies to understand more about the mechanisms driving the observed response of this species to a changing climate.

## Genome sequence report

**Figure 1.**
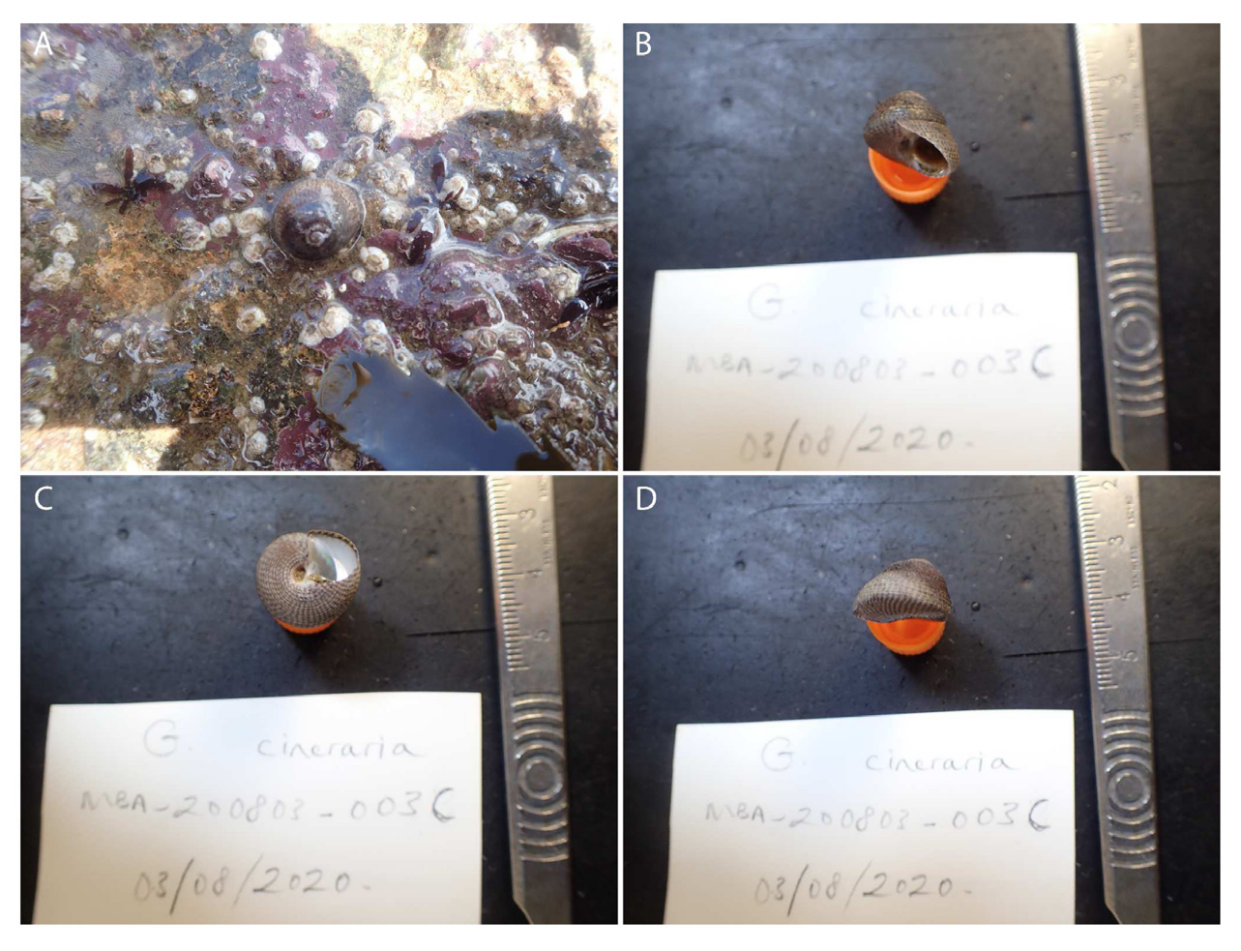
Image of the xgSteCine2 specimen. ( **A**) Image taken of the specimen prior to collection. ( **B**– **D**) Image of the shell of the specimen following preservation and processing.

The genome was sequenced from a single *S. cineraria* ( Figure 1) collected from Mount Batten, Devon, UK (latitude 50.36084, longitude -4.12833). A total of 42-fold coverage in Pacific Biosciences single-molecule long reads and 35-fold coverage in 10X Genomics read clouds were generated. Primary assembly contigs were scaffolded with chromosome conformation Hi-C data. Manual assembly curation corrected 408 missing/misjoins and removed 70 haplotypic duplications, reducing the assembly size by 2.51% and the scaffold number by 53.835%, and increasing the scaffold N50 by 123.17%.

**Figure 2.**
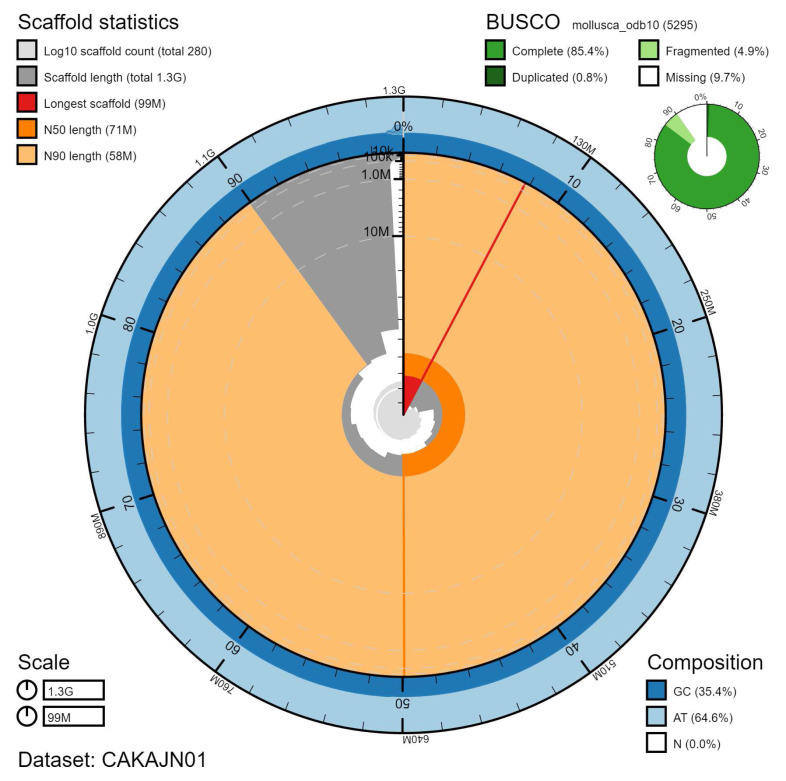
Genome assembly of *Steromphala cineraria*, xgSteCine2.1: metrics. The BlobToolKit Snailplot shows N50 metrics and BUSCO gene completeness. The main plot is divided into 1,000 size-ordered bins around the circumference with each bin representing 0.1% of the 1,270,504,078 bp assembly. The distribution of scaffold lengths is shown in dark grey with the plot radius scaled to the longest scaffold present in the assembly (98,775,408 bp, shown in red). Orange and pale-orange arcs show the N50 and N90 scaffold lengths (70,748,747 and 57,769,559 bp), respectively. The pale grey spiral shows the cumulative scaffold count on a log scale with white scale lines showing successive orders of magnitude. The blue and pale-blue area around the outside of the plot shows the distribution of GC, AT and N percentages in the same bins as the inner plot. A summary of complete, fragmented, duplicated and missing BUSCO genes in the mollusca_odb10 set is shown in the top right. An interactive version of this figure is available at https://blobtoolkit.genomehubs.org/view/Steromphala%20cineraria/dataset/CAKAJN01/snail.

The final assembly has a total length of 1,270 Mb in 283 sequence scaffolds with a scaffold N50 of 70.7 Mb ( Table 1). Of the assembly sequence, 99.23% was assigned to 18 chromosomal-level scaffolds (numbered by sequence length) ( Figure 2– Figure 5; Table 2). Large inversions between sister chromatids can be seen on chromosome 5 at 29.7–60.7 Mb and chromosome 11 at 17.7–39.7 Mb. Possible inversions are also seen on chromosome 11 at Mb 3.4–39.4 and 18–66 Mb. The assembly has a BUSCO v5.1.2 ( Manni *et al.*, 2021) completeness of 85.4% (single 84.6%, duplicated 0.8%) using the mollusca_odb10 reference set (n=5295). However, we believe that this relatively low BUSCO score is a result of limitations with the current mollusca_odb10 geneset. Using the metazoa_odb10 reference set (n=954), the assembly has a completeness of 97.6% (single 97.0%, duplicated 0.6%), which we believe is evidence of high completeness. While not fully phased, the assembly deposited is of one haplotype. Contigs corresponding to the second haplotype have also been deposited.

**Table 1.** Genome data for *Steromphala cineraria*, xgSteCine2.1.

*Project accession data*	
Assembly identifier	xgSteCine2.1
Species	*Steromphala cineraria*
Specimen	xgSteCine2
NCBI taxonomy ID	NCBI:txid216125
BioProject	PRJEB45667
BioSample ID	SAMEA7536348
Isolate information	Muscle
*Raw data accessions*	
PacificBiosciences SEQUEL II	ERR6939216, ERR6939217
10X Genomics Illumina	ERR6363284-ERR6363287
Hi-C Illumina	ERR6363289
PolyA RNA-Seq Illumina	ERR6688409
*Genome assembly*	
Assembly accession	GCA_916613615.1
*Accession of alternate haplotype*	GCA_916613985.1
Span (Mb)	1,270
Number of contigs	842
Contig N50 length (Mb)	6.2
Number of scaffolds	283
Scaffold N50 length (Mb)	70.7
Longest scaffold (Mb)	98.8
BUSCO * genome score	C:85.4%[S:84.6%,D:0.8%], F:4.9%,M:9.7%,n:5295

*BUSCO scores based on the mollusca_odb10 BUSCO set using v5.1.2. C= complete [S= single copy, D=duplicated], F=fragmented, M=missing, n=number of orthologues in comparison. A full set of BUSCO scores is available at https://blobtoolkit.genomehubs.org/view/Steromphala%20cineraria/dataset/CAKAJN01/busco.

**Figure 3.**
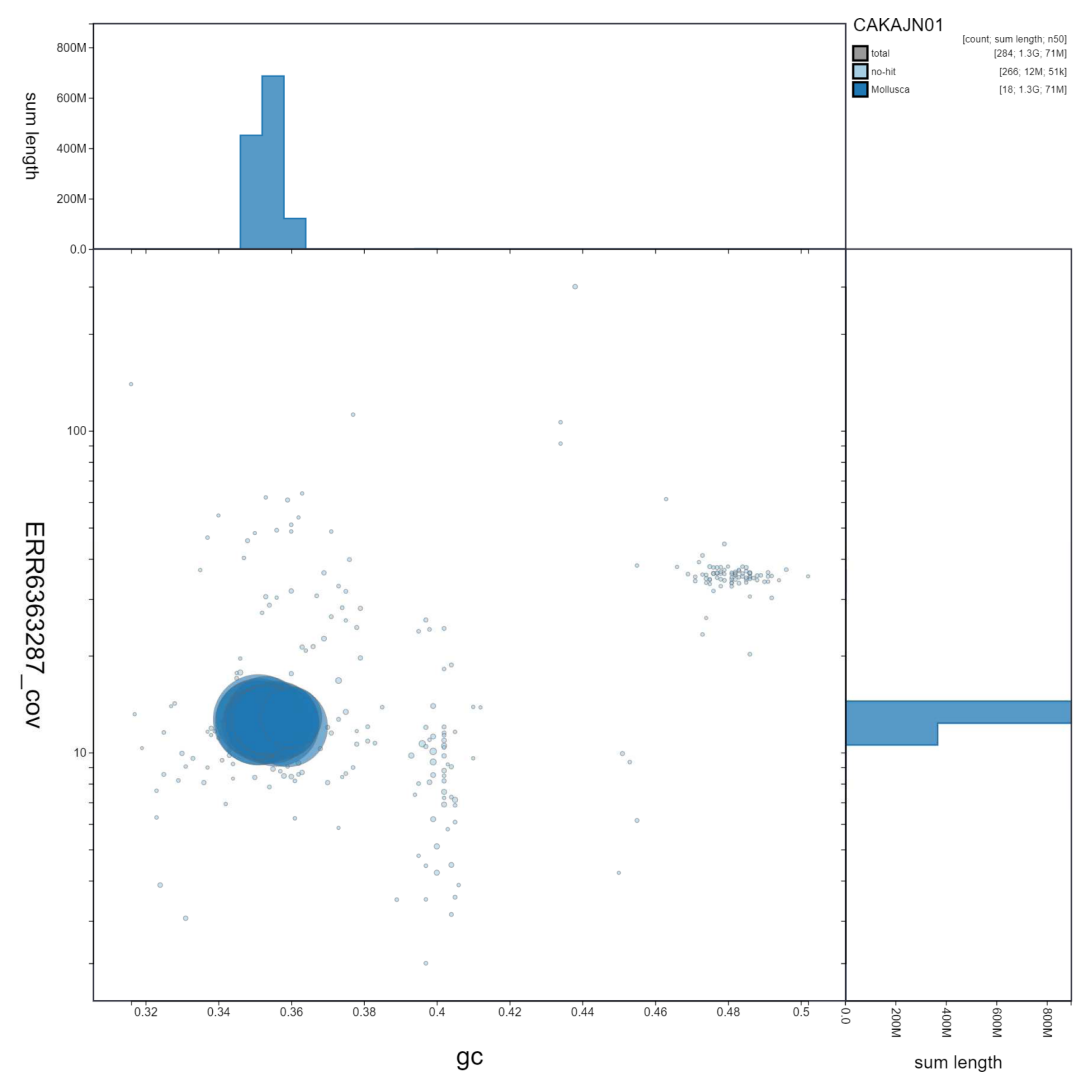
Genome assembly of *Steromphala cineraria*, xgSteCine2.1. GC coverage. BlobToolKit GC-coverage plot. Scaffolds are coloured by phylum. Circles are sized in proportion to scaffold length. Histograms show the distribution of scaffold length sum along each axis. An interactive version of this figure is available at https://blobtoolkit.genomehubs.org/view/Steromphala%20cineraria/dataset/CAKAJN01/blob.

**Figure 4.**
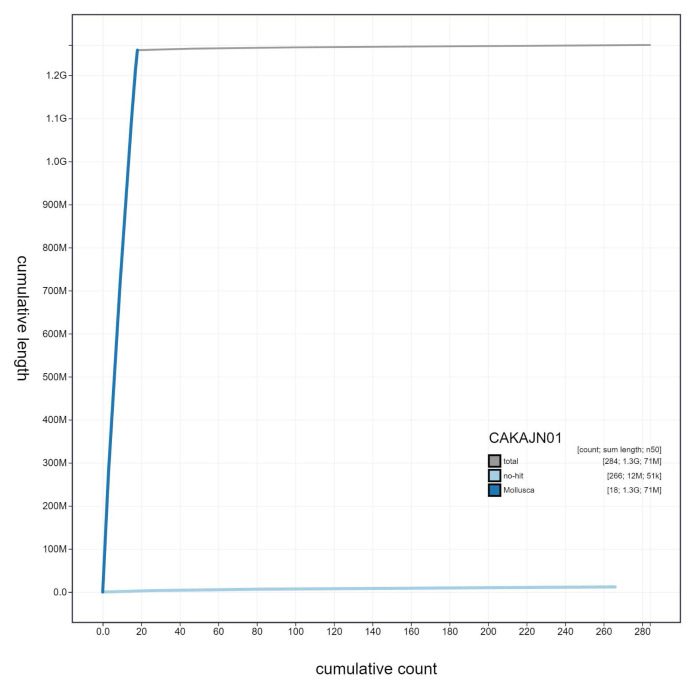
Genome assembly of *Steromphala cineraria*, xgSteCine2.1: cumulative sequence. BlobToolKit cumulative sequence plot. The grey line shows cumulative length for all scaffolds. Coloured lines show cumulative lengths of scaffolds assigned to each phylum using the buscogenes taxrule. An interactive version of this figure is available at https://blobtoolkit.genomehubs.org/view/Steromphala%20cineraria/dataset/CAKAJN01/cumulative.

**Figure 5.**
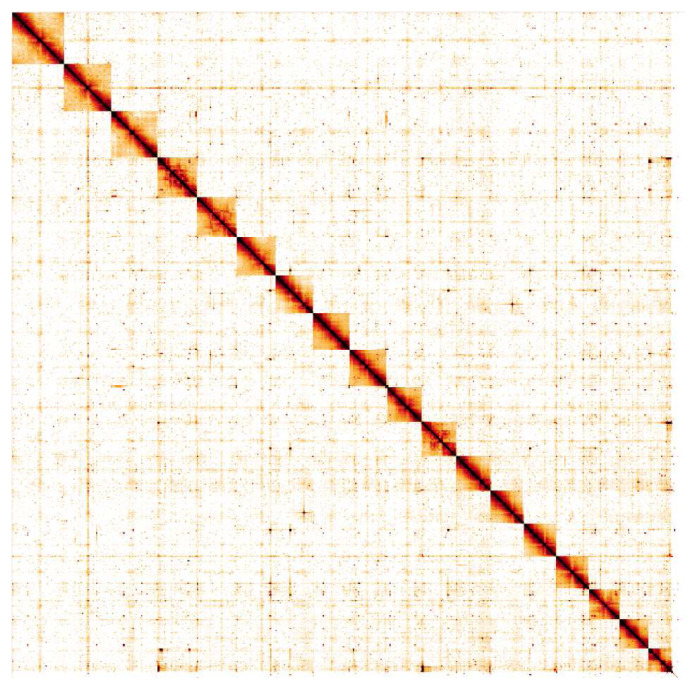
Genome assembly of *Steromphala cineraria*, xgSteCine2.1: Hi-C contact map. Hi-C contact map of the xgSteCine2.1 assembly, visualised in HiGlass. Chromosomes are shown in size order from left to right and top to bottom.

**Table 2.** Chromosomal pseudomolecules in the genome assembly of *Steromphala cineraria*, xgSteCine2.1.

INSDC accession	Chromosome	Size (Mb)	GC%
OU744720.1	1	98.78	35.1
OU744721.1	2	90.22	35.1
OU744722.1	3	87.49	35.6
OU744723.1	4	76.76	35.9
OU744724.1	5	75.56	35.5
OU744725.1	6	74.15	35.3
OU744727.1	7	70.75	35.0
OU744726.1	8	71.35	35.3
OU744728.1	9	70.17	35.7
OU744729.1	10	66.46	35.2
OU744730.1	11	66.15	35.5
OU744731.1	12	65.37	35.3
OU744732.1	13	62.64	35.1
OU744733.1	14	62.10	35.2
OU744734.1	15	61.92	35.4
OU744735.1	16	57.77	35.8
OU744736.1	17	56.52	35.3
OU744737.1	18	44.44	36.0
OU744738.1	MT	0.02	31.6
-	Unplaced	11.89	39.8

## Methods

### Sample acquisition and nucleic acid extraction

A single *S. cineraria* specimen (xgSteCine2) was collected from Mount Batten, Devon, UK (latitude 50.36084, longitude -4.12833) by Rob Mrowicki (Natural History Museum), Patrick Adkins and Joanna Harley (both Marine Biological Association), by hand. The samples were identified by the same individual and snap-frozen in liquid nitrogen.

DNA was extracted at the Tree of Life laboratory, Wellcome Sanger Institute. The xgSteCine2 sample was weighed and dissected on dry ice with tissue set aside for Hi-C and RNA sequencing. Muscle tissue was cryogenically disrupted to a fine powder using a Covaris cryoPREP Automated Dry Pulveriser, receiving multiple impacts. Fragment size analysis of 0.01–0.5 ng of DNA was then performed using an Agilent FemtoPulse. High molecular weight (HMW) DNA was extracted using the Qiagen MagAttract HMW DNA extraction kit. Low molecular weight DNA was removed from a 200-ng aliquot of extracted DNA using 0.8X AMpure XP purification kit prior to 10X Chromium sequencing; a minimum of 50 ng DNA was submitted for 10X sequencing. HMW DNA was sheared into an average fragment size between 12–20 kb in a Megaruptor 3 system with speed setting 30. Sheared DNA was purified by solid-phase reversible immobilisation using AMPure PB beads with a 1.8X ratio of beads to sample to remove the shorter fragments and concentrate the DNA sample. The concentration of the sheared and purified DNA was assessed using a Nanodrop spectrophotometer and Qubit Fluorometer and Qubit dsDNA High Sensitivity Assay kit. Fragment size distribution was evaluated by running the sample on the FemtoPulse system.

RNA was extracted from muscle tissue in the Tree of Life Laboratory at the WSI using TRIzol, according to the manufacturer’s instructions. RNA was then eluted in 50 μl RNAse-free water and its concentration RNA assessed using a Nanodrop spectrophotometer and Qubit Fluorometer using the Qubit RNA Broad-Range (BR) Assay kit. Analysis of the integrity of the RNA was done using Agilent RNA 6000 Pico Kit and Eukaryotic Total RNA assay.

### Sequencing

Pacific Biosciences HiFi circular consensus and 10X Genomics read cloud sequencing libraries were constructed according to the manufacturers’ instructions. Sequencing was performed by the Scientific Operations core at the Wellcome Sanger Institute on Pacific Biosciences SEQUEL II and Illumina NovaSeq 6000 instruments. Hi-C data were generated from additional muscle tissue of xgSteCine2 using the Arima v2.0 kit and sequenced on an Illumina NovaSeq 6000 instrument.

### Genome assembly

Assembly was carried out with Hifiasm ( Cheng *et al.*, 2021). Haplotypic duplication was identified and removed with purge_dups ( Guan *et al.*, 2020). One round of polishing was performed by aligning 10X Genomics read data to the assembly with longranger align, calling variants with freebayes ( Garrison & Marth, 2012). The assembly was then scaffolded with Hi-C data ( Rao *et al.*, 2014) using SALSA ( Ghurye *et al.*, 2019). The mitochondrial genome was assembled with MitoHiFi ( Uliano-Silva *et al.*, 2021), which performed annotation using MitoFinder ( Allio *et al.*, 2020). The assembly was checked for contamination and corrected using the gEVAL system ( Chow *et al.*, 2016) as described previously ( Howe *et al.*, 2021). Manual curation ( Howe *et al.*, 2021) was performed using gEVAL, HiGlass ( Kerpedjiev *et al.*, 2018) and Pretext. The genome was analysed within the BlobToolKit environment ( Challis *et al.*, 2020). Table 3 contains a list of all software tool versions used, where appropriate.

**Table 3.** Software tools used.

Software tool	Version	Source
Hifiasm	0.15	Cheng *et al.*, 2021
purge_dups	1.2.5	Guan *et al.*, 2020
SALSA2	3.0	Ghurye *et al.*, 2019
longranger align	2.2.2	https://support.10xgenomics.com/genome-exome/ software/pipelines/latest/advanced/other-pipelines
freebayes	v1.3.1	Garrison & Marth, 2012
MitoHiFi	2	https://github.com/marcelauliano/MitoHiFi
gEVAL	N/A	Chow *et al.*, 2016
HiGlass	1.11.6	Kerpedjiev *et al.*, 2018
PretextView	0.2.x	https://github.com/wtsi-hpag/PretextView
BlobToolKit	2.6.4	Challis *et al.*, 2020

### Ethics/compliance issues

The materials that have contributed to this genome note have been supplied by a Darwin Tree of Life Partner. The submission of materials by a Darwin Tree of Life Partner is subject to the Darwin Tree of Life Project Sampling Code of Practice. By agreeing with and signing up to the Sampling Code of Practice, the Darwin Tree of Life Partner agrees they will meet the legal and ethical requirements and standards set out within this document in respect of all samples acquired for, and supplied to, the Darwin Tree of Life Project. Each transfer of samples is further undertaken according to a Research Collaboration Agreement or Material Transfer Agreement entered into by the Darwin Tree of Life Partner, Genome Research Limited (operating as the Wellcome Sanger Institute), and in some circumstances other Darwin Tree of Life collaborators.

## Data availability

European Nucleotide Archive: Steromphala cineraria (grey top shell). Accession number PRJEB45667; https://identifiers.org/ena.embl/PRJEB45667.

The genome sequence is released openly for reuse. The *S. cineraria* genome sequencing initiative is part of the Darwin Tree of Life (DToL) project. All raw sequence data and the assembly have been deposited in INSDC databases. The genome will be annotated with the RNA-Seq data and presented through the Ensembl pipeline at the European Bioinformatics Institute. Raw data and assembly accession identifiers are reported in Table 1.

## Author information

Members of the Marine Biological Association Genome Acquisition Lab are listed here: https://doi.org/10.5281/zenodo.5913830.

Members of the Darwin Tree of Life Barcoding collective are listed here: https://doi.org/10.5281/zenodo.5744972.

Members of the Wellcome Sanger Institute Tree of Life programme are listed here: https://doi.org/10.5281/zenodo.5744840.

Members of Wellcome Sanger Institute Scientific Operations: DNA Pipelines collective are listed here: https://doi.org/10.5281/zenodo.5746904.

Members of the Tree of Life Core Informatics collective are listed here: https://doi.org10.5281/zenodo.5743293.

Members of the Darwin Tree of Life Consortium are listed here: https://doi.org/10.5281/zenodo.5638618.
