# Supplementary material for: The genome sequence of the grey top shell, Steromphala cineraria (Linnaeus, 1758)
Source: Wellcome Open Res. 2024 Mar 21;7:44. Originally published 2022 Feb 8. [Version 3] doi: 10.12688/wellcomeopenres.17677.3 (PMC11074690; doi:10.12688/wellcomeopenres.17677.3)
Supplement: Peer review history

# Reviewer report: Reviewer response for version 3

**DOI:** 10.21956/wellcomeopenres.23196.r77343

**Published:** 2024-05-06

**Authors:** André Gomes-dos-Santos

**Affiliations:** 1 Interdisciplinary Centre of Marine and Environmental Research, University of Porto, Porto, Portugal

The authors, Adkins et al., have addressed some of my previous concerns in their revised manuscript. However, not all of my concerns have been adequately addressed. I would appreciate the authors' opinions on the remaining issues I have raised. Since I did not receive a response to my previous review comments, I am uncertain as to why some of my concerns were not addressed in the revision.

 Nevertheless, I believe that the manuscript has been significantly improved.

Are sufficient details of methods and materials provided to allow replication by others?

Yes

Is the rationale for creating the dataset(s) clearly described?

Yes

Are the datasets clearly presented in a useable and accessible format?

Yes

Are the protocols appropriate and is the work technically sound?

Partly

Reviewer Expertise:

Bioinformatics, Phylogenomics, Genomics

I confirm that I have read this submission and believe that I have an appropriate level of expertise to confirm that it is of an acceptable scientific standard.

# Reviewer report: Reviewer response for version 3

**DOI:** 10.21956/wellcomeopenres.23196.r77344

**Published:** 2024-04-26

**Authors:** Taro Maeda

**Affiliations:** 1 Institute for Advanced Biosciences, Keio University, Mizukami, Japan

I am grateful for the opportunity to review the revised manuscript once again. I am pleased that the authors have provided clear information about the source samples for the RNA data. However, I have observed that some of my previous comments still need to be addressed in their comment or revised manuscript. Although I regret that I did not receive several corresponding comments from the author, Considering that this is a brief communication note, the information in question is not essential for this type of article format. If the editorial team determines to accept without the additional information,  I respect and agree with that decision.

 Below, I list the questions for which I could not find answers in the revised text or comments, along with my new comments on this version of the manuscript.

 ”The authors need to clarify the covering degree of their genome data against the total genome size and the number of chromosomes. Were the total scaffold length and the number of chromosomal pseudomolecules consistent with the predicted genome size and chromosome? At least the authors should describe the genome size predicted by the k-mer frequency analysis. If the authors did not measure the number of chromosomes, it should be made clear that they inferred the number of chromosomes from the Hi-C results.”

 "The authors opened PolyA RNA-seq results (ERR6688409), but it is unclear how this data contributed to the analysis. Although I expect it to be sequenced to predict gene regions, I found no description of the construction of the gene model. I also found no gene model data from the submitted genome data on the public database.

 The publication of raw RNA-Seq data is valuable for future analysis. However, the authors should clarify in the paper why this data was not used in the study."

 "A total of 42-fold coverage in Pacific Biosciences". Please clarify the predicted genome size of this species and the prediction method.

 What kind "-m" options were used for the gene model prediction for the BUSCO analysis? Did you use RNA-seq data during the BUSCO analysis? Please describe the number of gap regions (N-base) on the chromosomal-level scaffolds."

 The authors have responded as follows, but the corresponding link was not found in the text. 

 "We have included a link to the TOLQC page, which supplies required information about the genome sequencing and assembly, including k-mer frequency spectra analyses."

 When I investigated the TOLQC page, I believed the following page was relevant, but I could not find the corresponding link.

 https://tolqc.cog.sanger.ac.uk/darwin/molluscs/Steromphala_cineraria/

 Additionally, I could not find a genome size description based on k-mer analysis on the relevant web page.

Are sufficient details of methods and materials provided to allow replication by others?

Partly

Is the rationale for creating the dataset(s) clearly described?

Yes

Are the datasets clearly presented in a useable and accessible format?

Yes

Are the protocols appropriate and is the work technically sound?

Yes

Reviewer Expertise:

Sacoglossa, Genome, Symbiosis, Kleptoplasty

I confirm that I have read this submission and believe that I have an appropriate level of expertise to confirm that it is of an acceptable scientific standard.

# Reviewer report: Reviewer response for version 2

**DOI:** 10.21956/wellcomeopenres.19632.r50059

**Published:** 2022-05-04

**Authors:** Taro Maeda

**Affiliations:** 1 Institute for Advanced Biosciences, Keio University, Mizukami, Japan

I am honored to have the opportunity to review the paper by Adkins *et al*. This paper reports the genomic data of a sea snail, *Steromphala cineraria.* The genome of this species has never been revealed. There is no doubt that this data is worthy of publication. I think, however, some additional information needs to be provided to ensure the reliability of this data, as follows.

 Unfortunately, their genome data included no gene model and annotation information, and I found no description of how the RNA-Seq data contributed to the analysis. This deficiency may confuse the reader. Please clarify that the genomic data contain no gene model and annotation data and the role of RNA-Seq. 

**Abstract**

 The authors need to clarify the covering degree of their genome data against the total genome size and the number of chromosomes. Were the total scaffold length and the number of chromosomal pseudomolecules consistent with the predicted genome size and chromosome? At least the authors should describe the genome size predicted by the k-mer frequency analysis. If the authors did not measure the number of chromosomes, it should be made clear that they inferred the number of chromosomes from the Hi-C results.

**Table 1**

 The authors opened PolyA RNA-seq results (ERR6688409), but it is unclear how this data contributed to the analysis. Although I expect it to be sequenced to predict gene regions, I found no description of the construction of the gene model. I also found no gene model data from the submitted genome data on the public database.

 The publication of raw RNA-Seq data is valuable for future analysis. However, the authors should clarify in the paper why this data was not used in the study.

 Moreover, please describe whether this RNA-Seq data were obtained from the same individual used for genome sequencing. If the RNA is obtained from different samples, I recommend clarifying the sampling point and the preincubation state before the RNA extraction. 

**Genome sequence report**

 "A total of 42-fold coverage in Pacific Biosciences". Please clarify the predicted genome size of this species and the prediction method.

 What kind "-m" options were used for the gene model prediction for the BUSCO analysis? Did you use RNA-seq data during the BUSCO analysis? Please describe the number of gap regions (N-base) on the chromosomal-level scaffolds.

Are sufficient details of methods and materials provided to allow replication by others?

Partly

Is the rationale for creating the dataset(s) clearly described?

Yes

Are the datasets clearly presented in a useable and accessible format?

Yes

Are the protocols appropriate and is the work technically sound?

Yes

Reviewer Expertise:

Sacoglossa, Genome, Symbiosis, Kleptoplasty

I confirm that I have read this submission and believe that I have an appropriate level of expertise to confirm that it is of an acceptable scientific standard, however I have significant reservations, as outlined above.

# Reviewer report: Reviewer response for version 2

**DOI:** 10.21956/wellcomeopenres.19632.r49928

**Published:** 2022-04-14

**Authors:** André Gomes-dos-Santos

**Affiliations:** 1 Interdisciplinary Centre of Marine and Environmental Research, University of Porto, Porto, Portugal

Thank you for the opportunity to review this Data Note. In the Manuscript entitled " The genome sequence of the grey top shell, *Steromphala cineraria* (Linnaeus, 1758) ", the Authors Adkins et al, produced the high quality, chromosome level assembly of the grey top shell, using the de novo approach of the Darwin Tree of Life Project. The genome here presented has been made publicly available and is undoubtedly an important genomic resource. However, as seen in detail below, I have some concerns regarding possible technical issues. Thus, I am in favour of indexing, but only after the raised issues are answered.   

**Abstract**

 Please also include the overall stats of the mtDNA assembly in the abstract.

**Background**

 Regarding the species distribution, the authors report “Its geographical distribution ranges from southern Portugal and north to the White Sea in northern Russia, becoming rarer at its range edges as thermal limits are approached (Nekhaev, 2013).”

 However, in the Nekhaev 2013, the following is stated (please note that *Gibbula cineraria* is a synonym of Steromphala cineraria):

- “Distribution: In Atlantic *G. cineraria* is distributed from Morocco to Northern Norway [Fretter,Graham, 1977; Poppe, Goto, 1991]. It is common along the Norway coast, but live animals were not found in East Finnmark [Høisœter, 2009].”
- “Remarks: This species was previously reported from Ura Bay [Knipowitsch, 1900] but not mentioned in recent Russian faunistic and taxonomic literature [Galkin, 1955; Golikov, 1995; Golikovet al., 2001; Kantor, Sysoev, 2006].”

It is important that the authors revised this information accordingly to the citations they provided or any other recent citation that may support their claim.   

 Regarding the last sentence of the Background: “A high quality genome sequence for this species will allow future studies to understand more about the mechanisms driving the observed response of this species to a changing climate.”

 Although I recognize the fundamental importance of having a high-quality genome assembly to study such adaptative responses, by itself it is not enough. Especially given that no genome annotation is provided (which I understand is not a requirement). Given that, I would recommend changing the statement, so it is understood that the genome is important as it serves as a basal tool for future studies on the subject.

**Genome sequence report**

 Figure 1 - Images B-D have very poor resolution, the text on the labels is very hard to read. I recommend new photos.

 Regarding sequencing coverage, i.e., 42-fold in PacBio and 35-fold in 10X Genomics. Were these estimations based on any previous expectation of genome size? Have the authors produced a k-mer frequency spectrum analysis with the 10X Genomic reads? How did the authors decided the amount of sequencing output to produce? Molluscan genome sizes are highly variable, so it is customary to have guided assumptions prior to sequencing.

 Regarding the chromosome level scaffolding, given that SALSA does not require prior knowledge of the number of chromosomes and the high percentage of scaffolds assigned to chromosomes, the results seem highly reliable. However, I wonder if any prior estimation of the number of chromosomes for this group is available, that may further support the results.  

 Regarding the statement “Large inversions between sister chromatids can be seen on chromosome 5 at 29.7–60.7 Mb and chromosome 11 at 17.7–39.7 Mb. Possible inversions are also seen on chromosome 11 at Mb 3.4–39.4 and 18–66 Mb.” This is not evident from the Hi-C contact map. How did the authors determine this?

**Methods**

 The authors have produced a mitogenome assembly as well. However, they did not provide a characterization of the mitogenome nor have included an accession number for the mitogenome. According to Nekhaev (2013), identification based on morphological characters may be misleading for *Steromphala cineraria*. Since the whole mtDNA has proven to be a great complementary tool in Systematics, I think that is important to highlight this new resource as well. Please provide a general characterization of the mitogenome and include the accession number in Table 1.

 I noticed that the mtDNA available on NCBI (accession OU744738.1) is not annotated. I assume that it is not mandatory. As a note for the journal, I think that in the future, mtDNA annotation, which is very easily accomplished, should be a requirement. For instance, I noticed that this mtDNA sequence is almost 20.00bp long, largely due to a 2,853bp long tandem repeat of the motif TA. This repeat is not present in any of the other mtDNA available for the same genus (i.e., *Steromphala (Gibbula) umbilicaris*, accession NC_035682.1). Interestingly, the same pattern is present in another species of the same Family, i.e., *Phorcus lineatus *(accession OV121099.1), which is also associated with another Wellcome Sanger Institute Tree of Life project, also not annotated. Have the authors confirmed that this repetitive pattern is not a result of a sequencing error? Have the authors validated this pattern with both sequencing strategies?

 If it is not a sequencing error, then it is a novel character that may have phylogenetic implications and highlight the importance of using long read technologies to produce mtDNA assemblies. Please note that I limited my search to sequences on NCBI. Furthermore, I produced a quick annotation using Mitos2 web server, that resulted in the following warning “Translational exceptions: start=nad4, stop=nad4,nad4” which is reflected in the fact that the gene is apparently truncated. This was neither reported for the species of the same genus in NCBI (accession NC_035682.1) nor when I produced an annotation on Mitos2. I also annotated the *Phorcus lineatus* (accession OV121099.1) with Mitos2, which did not report such a warning. This, highlights the importance of producing such resources, given that if confirmed, it reveals a novel uncharacterized feature. On the other hand, it may provide additional small-scale validation of the overall quality of the genome assembly (given that the whole genome annotation is not provided). The fact that such an error might be present in the mtDNA may be a warning for similar errors in the whole genome.

Are sufficient details of methods and materials provided to allow replication by others?

Yes

Is the rationale for creating the dataset(s) clearly described?

Yes

Are the datasets clearly presented in a useable and accessible format?

Yes

Are the protocols appropriate and is the work technically sound?

Partly

Reviewer Expertise:

Bioinformatics, Phylogenomics, Genomics

I confirm that I have read this submission and believe that I have an appropriate level of expertise to confirm that it is of an acceptable scientific standard, however I have significant reservations, as outlined above.
